# Helminthofauna Diversity in Synanthropic Rodents of the Emilia-Romagna Region (Italy): Implications for Public Health and Rodent Control

**DOI:** 10.3390/vetsci11110585

**Published:** 2024-11-20

**Authors:** Filippo Maria Dini, Carlotta Mazzoni Tondi, Roberta Galuppi

**Affiliations:** Department of Veterinary Medical Sciences (DIMEVET), Alma Mater Studiorum University of Bologna, Via Tolara di Sopra 50, Ozzano dell’Emilia, 40064 Bologna, Italy; filippomaria.dini@unibo.it (F.M.D.); carlottamazzonitondi@gmail.com (C.M.T.)

**Keywords:** survey, helminths, synanthropic rodents, diversity, Emilia-Romagna region (Italy)

## Abstract

Synanthropic rodents are species well adapted to coexisting in anthropogenically influenced environments. This coexistence raises concerns about the potential risks of pathogen’s transmission due to their close proximity to human habitats. This study presents an epidemiological survey of the gastrointestinal helminth fauna in synanthropic rodents (*Mus musculus*, *Rattus rattus*, and *Rattus norvegicus*) from the Emilia-Romagna Region (Italy), aiming to provide updated data on the endoparasitic populations in these species. A total of 111 rodents, sampled from 2019 to 2021 during pest control programs, were examined for parasitic infections. Helminths were extracted through necropsy and microscopic analysis of gastrointestinal tracts and sediment, with species identification based on morphological characteristics. Overall, 72.1% of the rodents were found to be parasitized, with nematodes being the most prevalent. *Syphacia muris*, *Aspiculuris tetraptera*, *Nippostrongylus brasiliensis*, and *Heterakis spumosa* were the most frequently identified nematodes. Tapeworms, including *Rodentolepis nana* and *Hymenolepis diminuta*, were also detected, albeit in lower frequencies. The trematode *Brachylaima recurva* was recovered only in one *R. rattus*. Co-infection was common, particularly among rats, with 51.8% of black rats and 22% of brown rats harboring multiple parasitic species. Mice exhibited lower levels of polyparasitism, with only two individuals showing mixed infections. Interestingly, disparities between the detection of adult helminths and parasitic eggs were noted, especially in cases where no adults were observed, but eggs were found through sediment analysis. These findings suggest that traditional necropsy, especially with poorly preserved carcasses, may underestimate parasite prevalence. This highlights the importance of combining necropsy with microscopic techniques, such as flotation and sedimentation, for a more thorough assessment. Using these methods, nematodes with direct life cycles, such as *Syphacia spp.*, *Nippostrongylus brasiliensis*, and *Heterakis spumosa*, have been confirmed as widespread and cosmopolitan among rodent populations. The detection of zoonotic parasites raises concerns about potential transmission to humans, particularly in areas with poor sanitation and high rodent densities. These findings underscore the need for integrated rodent control and environmental sanitation to reduce zoonotic risks.

## Simple Summary

This study investigates gastrointestinal helminth infections in synanthropic rodents (house mice and two rat species) from Italy’s Emilia-Romagna region, in densely populated areas where these rodents live in close proximity to humans. Conducted between 2019 and 2021, the survey examined 111 rodents captured during pest control programs to identify parasitic worms in their gastrointestinal tracts. The findings revealed that 72.1% of the rodents were infected, with nematodes (roundworms) being the most common. Among the nematodes, *Syphacia muris*, *Aspiculuris tetraptera*, *Nippostrongylus brasiliensis*, and *Heterakis spumosa* were frequently detected, while tapeworms like *Rodentolepis nana* and *Hymenolepis diminuta* were found less often. This study also identified a rare trematode infection in a black rat. Infections with multiple parasite species were common, particularly in black and brown rats, though less frequent in mice. Notably, differences emerged between the detection of adult helminths and their eggs, suggesting that solely relying on necropsy may underestimate prevalence. Combining necropsy with microscopic sedimentation techniques provided a more accurate diagnosis. The presence of human-transmissible parasites emphasizes the importance of integrated rodent control and sanitation efforts to mitigate the risks of parasite transmission to humans, especially in areas where rodents are abundant and sanitation is poor.

## 1. Introduction

Rodent species commonly associated with human settlements, including the house mouse (*Mus musculus*, L. 1578), the black rat (*Rattus rattus*, L. 1578), and the brown rat (*Rattus norvegicus*, Berkenhout, 1769), have become globally distributed, primarily due to human transportation, and are well adapted to urban environments. These synanthropic rodents are frequently found in anthropogenic habitats and are widely regarded as pests, impacting both the agri-food and construction sectors, leading to significant economic losses annually [1]. Rodents are highly adaptable and have frequently accompanied human migrations, allowing them to establish and sometimes become invasive in new regions, often affecting local biodiversity and impacting human activities [2]. Their spread is particularly accelerated by global changes, such as urbanization and shifts in land use, which favor the expansion of rodent species due to their affinity for human-altered environments. With growing urban populations, these shifts are expected to drive significant ecological and public health changes associated with rodents [3,4]. As reservoir hosts for over 60 zoonotic diseases, rodents play a major role in disease transmission. High-impact diseases transmitted by rodents include salmonellosis, plague, leptospirosis, and hantavirus-related syndromes. They may also carry various pathogens such as *Mycobacterium* spp., *Escherichia coli*, and agents of tularemia, bartonellosis, and Lyme disease, underscoring their complex role in spreading infectious diseases across different settings, including both direct (or environmental), and vector transmission [5,6].

Ecologically, invasive rodents not only affect native species through direct predation and competition but also through indirect effects, such as introducing non-native parasites or increasing the prevalence of existing native parasites. From a public health perspective, rodents serve as key reservoirs for various zoonotic helminths, and while numerous rodent species can carry zoonotic parasites, most of these parasites have been identified in synanthropic rodent species like *Rattus* spp. *and Mus* spp. [7]. These include the cestodes *Rodentolepis nana* [8] and *Hymenolepis diminuta* [9], as well as the acanthocephalan *Moniliformis moniliformis* [10]. In Italy, the enteric parasite fauna of mice and rats has been minimally studied, with only a few investigations conducted in the past [11,12,13,14,15].

One of the challenges in conducting these investigations is that, unless “ad hoc” trapping campaigns are implemented, pest control companies typically use poisoned bait, making it difficult to recover the carcasses of the animals. The aim of this study was to perform an epidemiological survey of the helminth fauna in synanthropic rodents (house mice, black rats, and brown rats) within the provinces of Ferrara, Forlì-Cesena, and Ravenna in the Emilia-Romagna Region (ER). The study sought to characterize the distribution, abundance, and primary species of gastrointestinal parasites in these animals, with the objective of providing updated data on the endoparasite populations in rats and mice, thereby addressing the current lack of recent information on this topic.

## 2. Material and Methods

**Figure 1:**
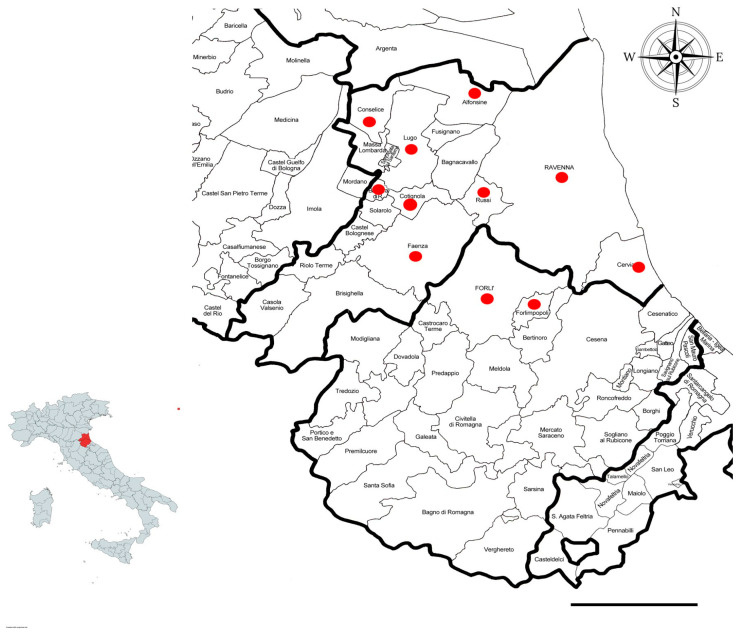
Different municipalities of the provinces of Ravenna and Forlì-Cesena in which the samplings were carried out. Scale bar: 20 km.

From June 2019 to June 2021, 111 synanthropic rodents were sampled during pest control programs in different municipalities in the provinces of Forlì-Cesena and Ravenna (ER) (Figure 1). The study area, located in the southeastern part of the Po Valley, is characterized by a highly urbanized plain. Specifically, the population density in Ravenna Province is approximately 209 inhabitants per square kilometer, and in Forlì-Cesena Province, it is about 166 inhabitants per square kilometer. All sampling was conducted by a specialized pest control company within or near urban areas. The sample included 27 black rats (*Rattus rattus*), 43 brown rats (*Rattus norvegicus*), and 41 mice (*Mus musculus*). The samples were collected by professional rodent control services and stored at −20 °C before processing.

During necropsy, the entire gastrointestinal tract of each rodent was collected and processed following the methodology described by Galbreath et al. [16]. The tract was carefully straightened and incised longitudinally from the posterior end using scissors with at least one rounded blade. Any large parasites present in the gastrointestinal contents were extracted, rinsed with deionized water, and preserved in 70% ethanol. The mucosal surface was scraped using a microscope slide, rinsed with water, and the resulting material was transferred into a conical container. The sediment was subjected to multiple washings to ensure thorough cleaning and was then examined under a stereoscope. Helminths were isolated, rinsed with deionized water, and fixed in 70% ethanol for further analysis.

**Figure 2:**
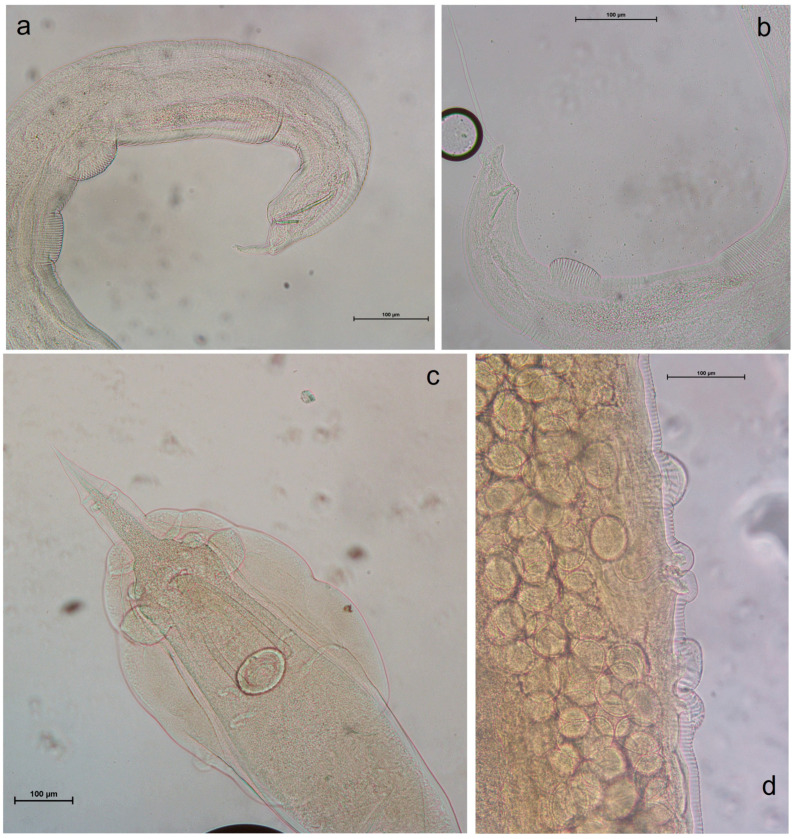
Anatomical details of *Syphacia* spp. and *Heterakis spumosa* in *R. norvegicus* and *M. musculus.* (**a**) *Syphacia obvelata*, male, tail; (**b**) *S. muris*, male, tail; (**c**) *Heterakis spumosa*, male, tale; (**d**) *H. spumosa*: female, vulvar opening.

**Figure 3:**
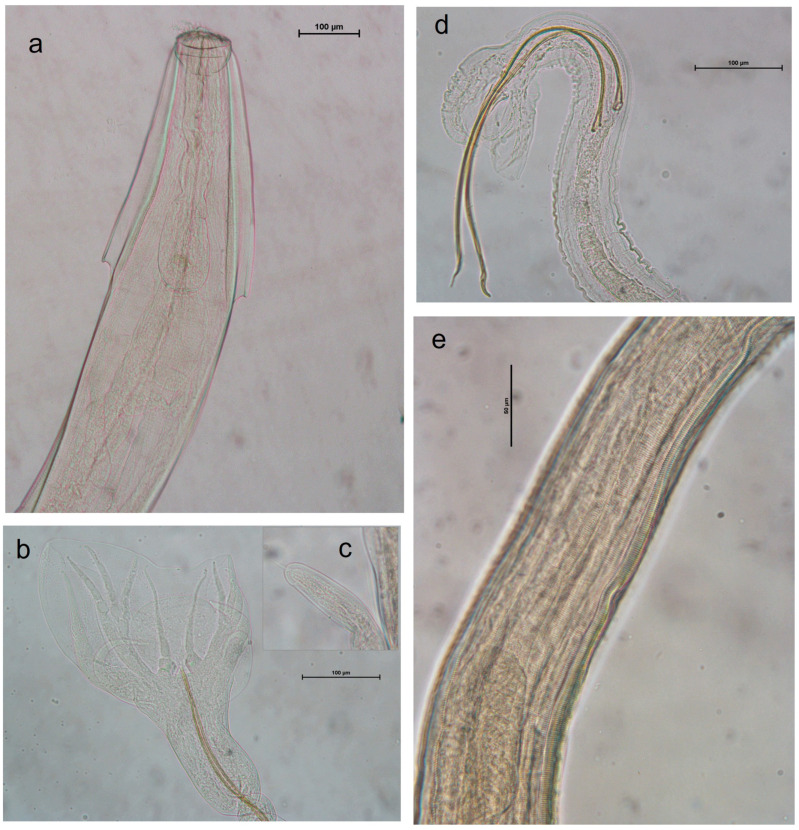
Anatomical details of *Aspiculuris tetraptera* and Strongylda from *R. norvegicus*. (**a**) *Aspiculuris tetraptera*, head; (**b**) *Heligmosomoides polygyrus*, male tail; (**c**) *H. polygyrus*, female tail; (**d**) *Nippostrongylus brasiliensis*, male tail; (**e**) *N. brasiliensis*, horizontal striations and longitudinal ridges.

**Figure 4:**
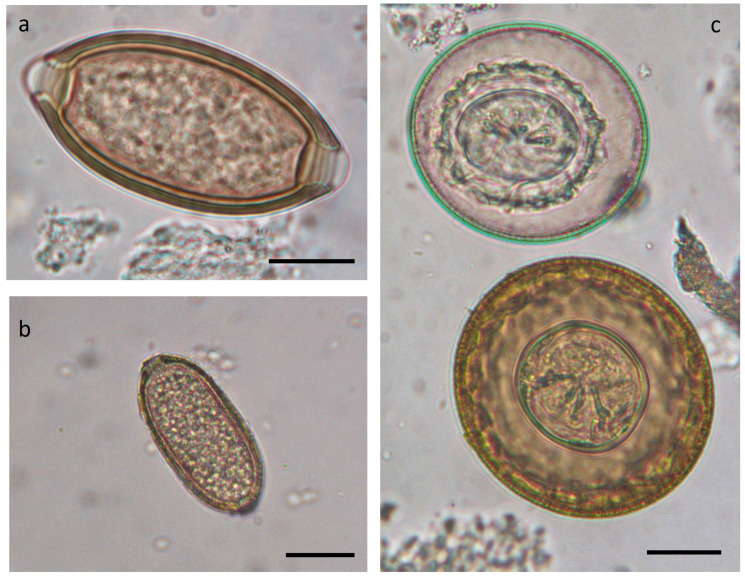
Eggs found at microscopic examination of sediment of both *Rattus* spp. and *M. musculus*. (**a**) *Trichuris* sp.; (**b**) *Capillaria* spp.; (**c**) *Rodentolepis nana* (upward), and *Hymenolepis diminuta* (down). Scale bar: 20 µm.

The sediment remaining after the removal of adult helminths was further processed to detect parasitic eggs that were not visible under the stereomicroscope. Both the sediment and the floated material, prepared using a solution with a specific gravity of 1.300, were examined under a light microscope. All collected helminths were subsequently mounted on slides in lactophenol for microscopic observation and identification (Figure 2, Figure 3 and Figure 4), following descriptions and identification keys from the literature. Observations were performed using a Leica DMLS optical microscope (Leica Microsystems Srl, Milan, Italy). Measurements and images were captured using a NIKON DS-Fi2 digital camera in conjunction with NIS Elements NIKON 4.10.01 image acquisition software.

Data on the analyzed animals and the parasites identified were recorded in an Excel database. A descriptive analysis was performed to calculate the frequencies of the various variables. For each identified parasitic species, prevalence, mean abundance, mean infestation intensity, and sex ratio were determined. The concordance between the presence of adult helminths and the microscopic examination of intestinal content after flotation was assessed using Cohen’s kappa coefficient. Pearson’s χ^2^ test was employed to evaluate the association between parasitological findings and factors such as the area of origin, rodent species, and sex. Differences were considered statistically significant at *p* < 0.05.

## 3. Results

Out of 111 rodents examined, 89 (80.2%) were collected in the province of Ravenna (RA) and the remaining 22 (19.8%) were collected in the province of Forlì-Cesena (FC). Among these, 53.2% were male and 46.8% were female.

Eighty rodents (72.1%) tested positive for parasitic infection at necropsy. Table 1 presents the species examined, the area of capture, and their positivity to infection with nematodes, tapeworms, trematodes, and multiple parasitic infections.

In total, 27 gastrointestinal tracts from *Rattus rattus* were examined, comprising 11 males and 16 females. Table 2 summarizes the data on the parasitic species found in this host. *Syphacia muris* was the most frequently encountered species (59.3%), followed closely by *Aspiculuris tetraptera* (55.6%). In 12 subjects (44.5%), these two parasitic species were present together in the same individuals. *Heterakis spumosa*, *Nippostrongylus brasiliensis*, *Eucoleus gastricus*, and *Brachylaima recurva* were each found in only one subject (3.7%). Six black rats tested positive for tapeworm infestations: three were positive for *Rodentolepis nana* (11.2%), one for *Rodentolepis straminea* (3.7%), and two for *Hymenolepis diminuta* (7.4%). The microscopic features of *Syphacia muris* and *Heterakis spumosa* can be observed in Figure 2.

Forty-three gastrointestinal tracts were obtained from carcasses of *Rattus norvegicus*, comprising 27 males and 16 females. Table 3 summarizes the data regarding the parasitic species found. The most frequently encountered species were *Heterakis spumosa* (60.5%) and *Nippostrongylus brasiliensis* (55.8%). In 18 subjects (41.9%), these two species coexisted. *Syphacia muris* and *Aspiculuris tetraptera* were found with frequencies of 14% and 4.7%, respectively, while *Heligmosomoides polygyrus* and *Eucoleus gastricus* were each found in only one subject. Regarding tapeworms, only six samples (14%) tested positive for *Hymenolepis diminuta*. The microscopic features of *Aspiculuris tetraptera* and Strongylida recovered in *R. norvegicus* can be observed in Figure 3.

A total of 41 gastrointestinal tracts of *Mus musculus* were analyzed, comprising 21 males and 20 females. Approximately 50% of these individuals showed the presence of adult helminths. Table 4 summarizes the data on the parasitic species found. The most frequently encountered helminth was *Syphacia obvelata* (39%), while the other species were found in a limited number of individuals.

The simultaneous presence of two or three parasitic species was observed in many hosts during intestinal content macroscopic analysis. Specifically, 51.8% of the examined *Rattus rattus* were found to be polyparasitized, with 85.7% of these showing co-infection of *Syphacia muris* and *Aspiculuris tetraptera*. In *Rattus norvegicus*, the predominant co-infection involved *Heterakis spumosa* and *Nippostrongylus brasiliensis*, accounting for 77.3% of the 22% polyparasitized host. Conversely, in *Mus musculus*, only two subjects were polyparasitized: one with *Syphacia obvelata* and *Nippostrongylus brasiliensis*, and the other with *Syphacia muris* and *Heterakis spumosa*. Other parasitic associations were also noted, but with considerably lower percentages (see Table 5).

Out of the 111 gastrointestinal contents analyzed through microscopic examination by sedimentation and flotation, 47 (42.3%) tested positive for helminth eggs. Among these, five (10.6%) contained only *Trichuris* sp. eggs, seven (14.9%) *Eucoleus* sp. eggs, two (4.3%) Ascarids eggs, five (10.6%) pinworms eggs, and one (2.1%) strongyles eggs. Cestode oophores attributable to *Rodentolepis nana* and *Hymenolepis diminuta* were found together in five (10.6%) samples. The remaining samples contained eggs from various species found in association (Table 6). Details of the eggs’ morphology can be observed in Figure 4.

The comparison of the results obtained with the two methods (collection of adult helminths or microscopic observation of eggs after sedimentation/flotation) with respect to the different orders of parasites is showed in Table 7.

As regards the pinworms (Oxyurida: *Syphacia muris*, *Syphacia obvelata, Aspiculuris tetraptera*), only 4 out of 47 (8.5%) of the subjects who presented adults in the intestine were also positive for eggs at microscopic examination, while in 6 out of 64 (9.4%) it was possible to observe the presence of eggs in the absence of the finding of adult helminths.

As regards the Strongylida (*Nippostrongylus brasiliensis* and *Heligmosomoides polygyrus*), 2 out of 29 positives for the presence of adults were positive by copromicroscopy (6.8%); on the contrary, 2 out of 82 samples (2.4%) in which no adults were observed tested positive at microscopic examination. Concerning Enoplida, only two samples were found to be positive for *Eucoleus* sp. both for eggs and adult helminths. A single sample was positive for *Trichuris* sp. at both tests, while 29 out of 108 samples in which the presence of adult parasites was not found were positive for trichurid eggs at the microscopic examination. On the contrary, concerning Ascaridida, 14 of 27 samples positive for adult helminths (51.9%) were positive for eggs after flotation, while only one was positive at microscopic examination in the absence of adult helminths in the intestine (1.2%). As regards the tapeworms (Cyclophillida: *Hymenolepis diminuta*. *Rodentolepis nana*), 10 subjects, out of 12 positives for adult helminths, were also positive at the microscopic examination (83.4%), while in 7 subjects out of 99 (7.6%) macroscopically negative for tapeworms were positive at sedimentation/flotation.

Considering the results obtained with both methods (search for adults or microscopic examination after sedimentation/flotation), which identify the total number of parasitized individuals overall, the two species of rats were found to be significantly more frequently infested (88.9% *R. rattus*, 93% *R. norvegicus*) than mice (51.2%) (Rr vs. Mm χ2y = 8.71 *p* = 0.0032; Rn vs. Mm χ2y = 16.40; *p* = 0.0001; Rn vs. Rr = not significant); conversely, there are no significant differences in the χ2 test in the number of males and females positive for parasites in the three rodent species examined.

## 4. Discussion

*Rattus* spp. and *Mus musculus* are species well adapted to coexisting in anthropogenically influenced environments. This coexistence raises concerns about potential human health risks due to their close proximity to human habitats. In recent years. attention in Italy has focused on the presence of zoonotic agents in rodent hosts such as *Leishmania* sp. [17], *Toxoplasma gondii* [18], Hepatitis E virus [19], and other viral pathogens [20]. However, the helminthic fauna and the potential zoonotic helminths associated with these rodents have not been thoroughly investigated, with only a few studies addressing endoparasites.

Indeed, one of the main limitations in studying the helminth fauna of synanthropic rodents is the difficulty in obtaining adequate samples. This challenge may explain why, despite the widespread distribution of these rodents and their close contact with humans, only four additional studies have been conducted on this topic in Italy from 1966 to the present, primarily in local literature (Table 8). Moreover, previous studies have only observed *R. rattus* in southern Italy and *R. norvegicus* in central regions. This study is the first to examine both *Rattus* species across the country.

Another potential limitation in studies involving omnivorous host species is the possibility that some parasites found in the rodents’ intestines could be present due to scavenging behavior, rather than true infection. This may lead to slight overestimations in the reported prevalence and abundance data. However, as all helminths identified in this study were rodent-specific, the likelihood of pseudoparasites introduced via scavenging can be ruled out

The gastrointestinal helminth fauna of *Rattus rattus*, *Rattus norvegicus*, and *Mus musculus* observed in this survey showed a predominance of nematode parasites. This observation aligns with previous studies conducted in the same regions (Table 8) and in other geographic areas [7,21,22,23,24] The prevalence of nematodes is likely attributed to their simple and direct life cycle, which facilitates their widespread distribution through their hosts [25].

Among rodent nematodes, the Oxyurida (genera *Syphacia* and *Aspiculurus*) have a rapid life cycle and a direct transmission route, typically occurring through the ingestion of embryonated eggs shed in feces. Additionally, retroinfection can occur when hatched larvae migrate from the anus back to the colon [26]. Due to their efficient mode of transmission, these parasites have a cosmopolitan distribution and are frequently found in laboratory mice and rats [27,28,29,30].

*S. obvelata* and *S. muris* are the elective species found in mice and rats, respectively, although cross-infections can occur [26,31], as evidenced in this study by a mouse testing positive for *S. muris*. Their presence has been previously reported in Italy [14,15] and the Mediterranean basin [32]. In this survey, these parasites were detected in 39% of mice and 31.5% of rats. In other regions, *S. obvelata* has been reported in synanthropic rats, with a low prevalence of less than 1.1%, though *S. muris* was absent [22,33]. Notably. *S. obvelata* is potentially zoonotic, as it was identified in a Bohemian child living in the Philippines [34].

*Aspiculuris tetraptera* is another Oxyuridae commonly found in the cecum and colon of various species of laboratory and wild mice, though it is rarely reported in rats [27]. However, the present study revealed an inverse trend: all positive samples belonged to the genus *Rattus*, specifically 15 *R. rattus* and two *R. norvegicus*, with no *Mus musculus* specimens testing positive. It is possible that some mice were false negatives; for instance, immature forms of the parasite have been reported to reside between the epithelium and the basal membrane of the colon, which might not have been detected by the scraping method used to collect the samples. The presence of *Aspiculuris* is also reported in the literature to be negatively affected by co-infection with other intestinal parasites. Specifically, *S. obvelata* has been shown to enhance host resistance to *Aspiculuris* [26]. In the present study, 39% of the analyzed mice tested positive for *S. obvelata*, which may have influenced the development of *A. tetraptera*.

Another nematode frequently recovered in our specimens was *Heterakis spumosa* (Ascaridida), a cosmopolitan parasite that infects rats and has been documented in various regions worldwide [7,35]. Its life cycle is direct, involving the ingestion of eggs that must mature in the external environment [36]. This may explain why *H. spumosa* is relatively rare in laboratory rats compared to Oxyuridae [27]. In the literature, *H. spumosa* has been rarely reported in *Rattus rattus* in Sicily and other Mediterranean islands [15]. However, it has been observed up to 69% of *Rattus norvegicus* in Tuscany and Emilia-Romagna [11,13], consistent with the present study where this nematode was found with high prevalence in *R. norvegicus,* and a significant number of parasites collected (667 specimens).

In our survey, *Heterakis spumosa* exhibited a prevalence of 60.5% and an abundance of 15.4 in *Rattus norvegicus*, ranking second only to *Nippostrongylus brasiliensis* (Trichostrongyloidea. Heligmonellidae), which had a prevalence of 56.3% and an abundance of 17.3. *N. brasiliensis* is a common small-intestinal parasite of *R. norvegicus* with a worldwide distribution and can also infrequently infect *Rattus rattus* and, even more rarely, *Mus musculus* [27,37], consistent with the findings of this study. *N. brasiliensis* has a direct life cycle, with infective larvae (L3) developing in the environment. This may explain its rarity in laboratory rodents, except in facilities with inadequate sanitation and management, similarly to *Heligmosomoides polygyrus* (formerly *Nematospiroides dubius*) [27]. *H. polygyrus* and *N. brasiliensis* were not previously described in Italy but have been reported in some Mediterranean islands [15]. In this study, *H. polygyrus* was found in only one *R. norvegicus*.

Among the Trichuridae, *Trichuris* sp. has been previously described in *Rattus rattus* in Sicily and other Mediterranean islands [15]. Similarly, *Eucoleus* (formerly *Capillaria*) has been reported only in *Rattus norvegicus* in the Emilia-Romagna region [13]. In the present survey, only two specimens—one *Rattus rattus* and one *Rattus norvegicus*—were found positive for the adult form of *Eucoleus gastricus* (syn. *Capillaria gastrica*). In contrast, *Eucoleus* sp. eggs were observed in 20 other subjects following microscopic examination of the sediment. Similarly, the adult form of *Trichuris muris* was found exclusively in mice. Trichurid eggs were detected in 12 subjects (11 *Rattus* spp. and one *Mus musculus*). Both parasites have a direct life cycle, with the infective L1 stage developing within the eggs, which are highly resistant to environmental conditions. This resilience may explain why, in most cases, the presence of eggs was not accompanied by the detection of adult worms. This discrepancy may be attributed to the poor condition of the gastrointestinal tracts: the inability to freeze the carcasses immediately after the rodents’ death led to rapid putrefaction and self-digestion, which likely damaged the adult parasites, making them more difficult to detect. In contrast, the eggs, being more resistant, were less affected by tissue decomposition.

A similar scenario has been observed with tapeworms. In the current study, 19 (17.1%) samples tested positive for tapeworms: 17 were confirmed through microscopic examination of the sediment, and among these, adult forms (often only segments of the parasite, with scolices frequently absent) were found in only 10 samples. In two samples, only adult forms were detected. It is likely that the poor condition of the carcasses hastened the degradation of adult specimens, leading to their absence in some cases, or partial degradation in others, particularly the loss of scolices, which made it difficult to accurately assess the abundance and intensity of tapeworm infestation. Notably, tapeworm eggs were more effectively detected by microscopic examination of intestinal sediment, while flotation techniques, although capable of detecting eggs, often resulted in damage to their walls due to the use of the 1300 solution, complicating egg measurement.

Two species from the family Hymenolepididae were identified in the rodents examined: *Rodentolepis nana* (syn. *Hymenolepis nana, Hymenolepis fraternal, Vampirolepis nana*) and *Hymenolepis diminuta* (more frequently detected). Species identification, in the absence of well-preserved adult specimens, was based on egg morphology [38,39]. These species have previously been reported in mice and rats in Italy and the Mediterranean islands [11,13,14,15].

*Rodentolepis nana* and *H. diminuta* are both of zoonotic concern. The former, with various rodent species serving as definitive and reservoir hosts [40,41], can follow either an indirect life cycle—utilizing grain beetles (*Tenebrio* spp.) or fleas as intermediate hosts—or a direct life cycle. In the direct cycle, eggs are excreted into the environment via feces and may be ingested by other rodents or humans [42,43]. Additionally, in both species. self-infection can occur within the definitive host, wherein eggs hatch in the small intestine of the adult’s host, with metacestodes developing into mature worms without leaving the host [40]. *Rodentolepis nana* has a cosmopolitan distribution and is likely the most common tapeworm causing zoonotic infections globally, particularly among children living in poor sanitary conditions [41]. Humans can also act as reservoirs, and horizontal transmission between humans can occur through hand-to-mouth contact in settings with inadequate hygiene [44,45]. *Hymenolepis diminuta*, with *Rattus norvegicus* as its definitive host and *Tenebrio molitor* as its intermediate host, follows only an indirect life cycle. Human infection occurs accidentally through the ingestion of infected insects containing cysticercoids. This indirect transmission may explain why human hymenolepiasis caused by *H. diminuta* is less common than that caused by *R. nana* [46], despite being reported in 80 countries from 1810 to 2018 [9].

Identifying and monitoring the presence of these parasites in a given area is essential for understanding the potential risks to human health. Implementing measures to control rodent populations, for instance, can help mitigate the spread of these tapeworms and reduce the likelihood of human infection outbreaks.

With respect to trematodes, *Brachylaima recurva* (syn. *Heterolope aequans*), belonging to the family Brachylaimidae, was found in the gastrointestinal tract of a single specimen of *R. rattus*. This parasite has an indirect, and entirely terrestrial, life cycle that requires two intermediate hosts, both of which are land snails. The definitive hosts are numerous and include mammals, birds, reptiles, and amphibians. Humans can act as accidental hosts by ingesting raw, infected snails [47].

Initially, human infection was thought to be limited to specific cases: in children, through deliberate or playful ingestion of snails, and in adults, through accidental ingestion of snails present on poorly washed vegetables. However, in some regions, it has been demonstrated that human infection mainly occurs through the consumption of snails as food. For instance, a study found a high prevalence of this parasite in snails sold as food in the Spanish city of Tudela, with the highest infection rates occurring during the autumn season [48].

Macchioni in 1967 [12] described *B. recurva* as a “rare” trematode in Italy, a finding consistent with our results, which identified *Brachylaima* in only one specimen. This rarity likely contributes to the limited presence of *B. recurva* in the literature as a zoonotic agent.

This study’s examination of gastrointestinal parasites in rats and mice not only revealed the diversity of helminths present but also underscored the concept of polyparasitism (the simultaneous occurrence of multiple parasites within the same host). Wild populations often harbor multiple parasite species [49]. Co-infection can result from ecological and behavioral factors, pathogen competition, and their localization within the host, while the host’s immune response may also play a role in determining the likelihood of co-infection [23].

For example, *Heligmosomoides polygyrus* is known to increase susceptibility to other intestinal helminth infections [50]. Keeling in 1961 [51] demonstrated that mice with natural pinworm infections exhibit reduced susceptibility to *Trichuris muris*, but their susceptibility increases after pinworm removal. Additionally, when *Aspiculuris* sp. co-occurs with an established *Trichuris* infection, the *Aspiculuris* burden is significantly reduced.

In rodents, the negative interaction between *Syphacia muris* and *T. muris* likely results from competition between these parasites. In contrast, co-infection with *Nippostrongylus brasiliensis* and *S. muris* seems more compatible, as they inhabit different regions of the intestine and do not directly compete [23]. In the current study, specific parasite associations were observed, particularly the co-infection of *S. muris* and *Aspiculuris tetraptera* in *Rattus rattus* and *Heterakis spumosa* and *N. brasiliensis* in *Rattus norvegicus*.

## 5. Conclusions

The analysis of 111 gastrointestinal tracts from rodents in the Emilia-Romagna region revealed diverse and abundant helminth fauna among synanthropic rodents. Widespread nematodes such as *Syphacia* sp., *Nippostrongylus brasiliensis*, and *Heterakis spumosa* reflect the cosmopolitan distribution of these nematodes with direct life cycles. However, parasite detection may be underestimated due to carcass preservation issues, as shown by cases where only parasite eggs, not adults, were found. Thus, necropsy should be paired with sediment analysis using direct sedimentation and flotation techniques. The presence of zoonotic species highlights that inadequate sanitation and high rodent populations may establish zoonotic cycles, underscoring the need for rodent control and sanitation to reduce parasite transmission to humans.

## Figures and Tables

**Table 1:** Rodents examined and positivity to the various helminth taxa.

Species	N°	Sex	Origin: FC	Origin: RA	N° Positive for Nematoda (%)	N° Positive for Cestoda (%)	N° Positive for Trematoda (%)	N° Positive for Polyparasitism (%)
*R. rattus*	27	♂ 11 ♀ 16	4	23	20 (74.1%)	6 (22.3%)	1 (3.7%)	14 (51.8%)
*R. norvegicus*	43	♂ 27 ♀ 16	12	31	37 (86.0%)	6 (14%)	0	22 (51.2%)
*M. musculus*	41	♂ 21 ♀ 20	6	35	19 (46.3%)	0	0	2 (4.9%)
Total	111	♂ 59 (53.2%) ♀ 52 (46.8%)	22 (19.8%)	89 (80.2%)	76 (68.5%)	12 (10.8%)	1 (0.9%)	38 (34.2%)

**Table 2:** Adult helminths found in the gastrointestinal tracts of *Rattus rattus*.

Parasite Species	N. Positive (%)	Host Male N° (%)	Host Female N°(%)	Total Number of Helminths	Mean Intensity	Range	Abundance	Parasite Sex Ratio
*Nippostrongylus brasiliensis*	1 (3.7%)	1 (9.1%)	/	1	1	0–1	3.7	♂ 0 ♀ 1
*Heterakis spumosa*	1 (3.7%)	/	1 (6.3%)	1	1	0–1	3.7	♂ 0 ♀ 1
*Syphacia* *muris*	16 (59.3%)	6 (54.5%)	10 (62.5%)	200	12.5	1–30	7.4	♂ 8 ♀ 192
*Aspiculuris tetraptera*	15 (55.6%)	6 (54.5%)	9 (56.3%)	159	10.6	1–38	5.9	♂ 39 ♀ 120
*Eucoleus gastricus*	1 (3.7%)	1 (9.1%)	/	2	2	0–2	7.4	♂ 0 ♀ 2
*Rodentolepis nana*	4 (14.8%)	1 (9.1%)	3 (18.7%)	N.A.	N.A.	N.A.	N.A.	N.A.
*Hymenolepis diminuta*	2 (7.4%)	1 (9.1%)	1 (6.3%)	N.A.	N.A.	N.A.	N.A.	N.A.
*Brachylaima recurva*	1 (3.7%)	/	1 (6.3%)	1	1	0–1	3.7	N.A.

N.A.: not applicable. The number of cestodes usually is determined by the presence of the scolex, which is not always present. This missing data consequently affects the “mean intensity”, “range”, and “abundance” categories.

**Table 3:** Adult helminths found in gastrointestinal tract of *Rattus norvegicus*. N.A.: not applicable.

Parasite Species	N. Positive (%)	Host Male N° (%)	Host Female N°(%)	Total Number of Helminths	Mean Intensity	Range	Abundance	Parasite Sex Ratio
*Nippostrongylus brasiliensis*	24 (55.8%)	15 (55.5%)	9 (56.3%)	744	31	1–76	17.3	♂ 231 ♀ 513
*Heligmosomoides polygyrus*	1 (2.3%)	1 (3.7%)	/	13	13	5–8	0.3	♂ 8 ♀ 5
*Heterakis spumosa*	26 (60.5%)	16 (59.3%)	10 (62.5%)	666	25.6	1–94	15.5	♂ 288 ♀ 378
*Syphacia* *muris*	6 (14%)	4 (14.8%)	2 (12.5%)	58	9.7	1–28	1.3	♂ 7 ♀ 51
*Aspiculuris tetraptera*	2 (4.7%)	2 (7.4%)	/	20	9	1–12	0.4	♂ 14 ♀ 6
*Eucoleus gastricus*	1 (2.3%)	/	1 (6.3%)	4	4	0–4	0.1	♂ 0 ♀ 4
*Hymenolepis diminuta*	6 (14%)	5 (18.5%)	1 (6.3%)	N.A.	N.A.	N.A.	N.A.	N.A.

**Table 4:** Adult helminths found in gastrointestinal tract of *Mus musculus*. N.A.: not applicable.

Parasite Species	N. Positive (%)	Host Male N° (%)	Host Female N°(%)	Total Number of Helminths	Mean Intensity	Range	Abundance	Parasite Sex Ratio
*Nippostrongylus brasiliensis*	2 (4.9%)	1 (2.4%)	1 (5%)	9	4.5	1–4	0.2	♂ 4 ♀ 5
*Heligmosoimodes polygyrus*	2 (4.9%)	/	2 (10%)	18	9	3–9	0.4	♂ 9 ♀ 9
*Syphacia* *muris*	1 (2.4%)	/	1 (5%)	1	1	0–1	0.02	♂ ♀ 1
*Syphacia* *obvelata*	16 (39%)	10 (47.6%)	6 (30%)	127	7.9	1–38	3.1	♂ 21 ♀ 106
*Trichuris muris*	1 (2.4%)	/	1 (5%)	3	3	0–3	0.1	♂ 0 ♀ 3

**Table 5:** Polyparasitism in the rodents examined.

		Coinfections								
Host	N° Positive for Polyparasitism (%)	Sm + At (%)	Sm + At + Rn (%)	Sm + Hs + Hd (%)	Sm + At + Hs (%)	Hs + Nb (%)	Hs + Nb + Hd (%)	Hs + Nb + Hp (%)	So + Nb (%)	Sm + Hp (%)
*R. rattus*	14 (51.8%)	12 (85.7%)	3 (21.4%)	1 (7.1%)	/	/	/	/	/	/
*R. norvegicus*	22 (51.2%)	2 (9.1%)	/	/	1 (4.5%)	17 (77.3%)	2 (9.1%)	1 (4.5%)	/	/
*M. musculus*	2 (4.9%)	/	/	/	/	/	/	/	1 (50%)	1 (50%)

Legend: Sm: Syphacia muris. At: *Aspiculuris tetraptera*. Rn: Rodentolepis nana. Hs: Heterakis spumosa. Hn: *Hymenolepis diminuta*. Nb: *Nippostrongylus brasiliensis*. Hp: *Heligmosomoides polygyrus*. So: Syphacia obvelata.

**Table 6:** Samples with co-presence of eggs of different helminth species observed after flotation of intestinal contents.

Parasites	*E,A*	*E,A,C*	*E,O*	*E,O,C*	*E,T,A*	*E,T,A,S*	*E,C*	*A,C*	*T,C*	*T,E,C*	*T,A*	*T,O*	*O,C*
Samples positive (%)	4 (8.5%)	4 (8.5%)	1 (2.1%)	2 (4.3%)	1 (2.1%)	1 (2.1%)	2 (4.3%)	1 (2.1%)	1 (2.1%)	1 (2.1%)	2 (4.3%)	1 (2.1%)	1 (2.1%)

Legend: E: *Eucoleus* sp.. A: Ascarids. O: pinworms. C: cestoda (*Rodentolepis nana* and *Hymenolepis diminuta*). T: *Trichuris* sp.. S: strongilids (*Nippostrongylus brasiliensis*/*Heligmosomoides polygyrus*).

**Table 7:** Results obtained applying two different methods in the diagnosis of parasitic helminths of the synanthropic rodent.

Parasite Order	Test	Positive	Negative	Concordance	Khoen K
Strongylida	Macroscopic examination	29	82	73.9%	0.062
	Flotation of sediment	4	107		
Ascaridida	Macroscopic examination	27	84	87.4%	0.597
	Flotation of sediment	15	96		
Oxyurida	Macroscopic examination	47	64	55.8%	0.010
	Flotation of sediment	10	101		
Enoplida	Macroscopic examination	3	108	73.9%	0.128
	Flotation of sediment	32	79		
Cyclophillida	Macroscopic examination	12	99	91.9%	0.645
	Flotation of sediment	17	94		

**Table 8:** Helminths collected from gastrointestinal tracts of *R. rattus*. *R.. norvegicus,* and *M musculus* in Italy since 1966.

	[11,12]	[13]	[14]	[15]	Present Paper
Region of Italy	Tuscany and Emilia Romagna	Western Emilia Romagna	Western Sicily	Western Sicily	Eastern Emilia Romagna
***R. rattus*** **—n. examined**	**0**	**0**	**92**	**45**	**27**
*Nippostrongylus* *brasiliensis*	-	-	0	0	1 (3.7%)
*Heterakis spumosa*	-	-	0	1 (2.22%)	1 (3.7%)
*Syphacia muris*	-	-	38 (41.30%)	10 (22.22%)	16 (59.3%)
*Aspiculurus tetraptera*	-	-	3 (3.26%)	0	15 (55.6%)
*Mastophorus muris*	-	-	0	4 (8.89%)	0
*Eucoleus gastricus*	-	-	0	0	1 (3.7%)
*Trichuris* sp.	-	-	1 (1.08%)	0	0
*Hymenolepis nana*	-	-	2 (2.17)	0	3 (11.2%)
*Hymenolepis diminuta*	-	-	2 (2.17%)	7 (15.56%)	3 (11.1%)
*Brachylaima recurva*	-	-	15 (16.30%)	0	1 (3.7%)
***R. norvegicus*** **—n. examined**	**74**	**23**	**2**	**0**	**43**
*Strongyloides ratti*	31 (41.89%)	0	0	-	0
*Nippostrongylus* *brasiliensis*	0	14 (60.86%)	0	-	24 (55.8%)
*Heligmosomoides polygirus*	0	0	0	-	1 (2.3%)
*Heterakis spumosa*	25 (33.78%)	16 (69.56%)	0	-	26 (60.5%)
*Syphacia muris*	0	2 (8.69%)	0	-	6 (14%)
*Aspiculurus tetraptera*	1 (1.35%)	2 (8.69%)	0	-	2 (4.7%)
*Capillaria* sp.	0	1 (4.34%)	0	-	1 (2.3%)
*Hymenolepis nana*	13 (17.57%)	0	0	-	0
*Hymenolepis diminuta*	17 (22.97%)	16 (69.56%)	0	-	6 (14%)
*Echinostoma echinatus*	0	1 (4.34%)	0	-	0
***Mus musculus*** **—n. examined**	**51**	**0**	**6**	**44**	**41**
*Nippostrongylus* *brasiliensis*	0	-	0	0	2 (4.9%)
*Heligmosomoides polygirus*	0	-	0	0	2 (4.9%)
*Syphacia muris*	0	-	1 (16.67%)	0	1 (2.4%)
*Syphacia obvelata*	13 (25.49%)	-	0	10 (22.73)	16 (39%)
*Aspiculurus tetraptera*	4 (7.84%)	-	0	13 (29.55%)	0
*Protospirura muris*	3 (5.88%)	-	0	0	0
*Mastophorus muris*	0	-	0	5 (11.36%)	0
*Gongylonema musculi*	0	-	0	1 (2.27%)	0
*Trichuris muris*	0	-	0	4 (9.09%)	1 (2.4%)
*Hymenolepis nana*	4 (7.84%)	-	0	0	0
*Himenolepis diminuta*	0	-	0	1 (2.27%)	0
*Rodentolepis microstoma*	0	-	0	2 (4.55)	0
*Catenotaenia pusilla*	3 (5.88%)	-	0	1 (2.27%)	0
*Brachylaima* sp.	0	-	0	1 (2.77)	0

## Data Availability

The data that support the findings are available from the corresponding author.
